# Rapid recalibration of speech perception after experiencing the McGurk illusion

**DOI:** 10.1098/rsos.170909

**Published:** 2018-03-28

**Authors:** Claudia S. Lüttke, Alexis Pérez-Bellido, Floris P. de Lange

**Affiliations:** Radboud University, Donders Institute for Brain, Cognition and Behaviour, Nijmegen, The Netherlands

**Keywords:** perceptual learning, McGurk illusion, audiovisual integration, recalibration, signal detection theory

## Abstract

The human brain can quickly adapt to changes in the environment. One example is phonetic recalibration: a speech sound is interpreted differently depending on the visual speech and this interpretation persists in the absence of visual information. Here, we examined the mechanisms of phonetic recalibration. Participants categorized the auditory syllables /aba/ and /ada/, which were sometimes preceded by the so-called McGurk stimuli (in which an /aba/ sound, due to visual /aga/ input, is often perceived as ‘ada’). We found that only one trial of exposure to the McGurk illusion was sufficient to induce a recalibration effect, i.e. an auditory /aba/ stimulus was subsequently more often perceived as ‘ada’. Furthermore, phonetic recalibration took place only when auditory and visual inputs were integrated to ‘ada’ (McGurk illusion). Moreover, this recalibration depended on the sensory similarity between the preceding and current auditory stimulus. Finally, signal detection theoretical analysis showed that McGurk-induced phonetic recalibration resulted in both a criterion shift towards /ada/ and a reduced sensitivity to distinguish between /aba/ and /ada/ sounds. The current study shows that phonetic recalibration is dependent on the perceptual integration of audiovisual information and leads to a perceptual shift in phoneme categorization.

## 1. Introduction

A challenge in understanding speech is that we have to categorize complex ambiguous sounds into discrete percepts (e.g. phonemes /b/ and /d/). Different speakers can pronounce phonemes differently, but our brain can flexibly adapt to these instabilities, for instance, by taking into account visual information. Seeing someone speak can give useful information about what is said. Not only is this visual information used in understanding speech, there can even be a remapping how the sound is later perceived. In phonetic recalibration, how an ambiguous sound (artificially created so that it falls in between /b/ and /d/) is perceived, is determined by the recent audiovisual experience, i.e. whether the sound was presented together with a video of a speaker pronouncing /b/ or /d/ [1]. In other words, an ambiguous sound is disambiguated by the visual context which leads to a shift in boundaries between phonemes that persists after the visual context has ceased. Another example of phonetic recalibration occurs after the McGurk illusion, where conflicting auditory (/aba/) and visual (/aga/) inputs presented together can lead to a different percept (ada) [2]. This illusion results in a subsequent recalibration of the phonetic boundaries, as it affects how the syllable /aba/ is later categorized [1,3].

In the present work, we address several outstanding questions regarding the characteristics and underlying mechanisms of phonetic recalibration. Firstly, while most phonetic recalibration studies assume that simple exposure to audiovisual speech conflict can result in a recalibration [4–6], it is unclear whether the audiovisual information needs to be actually integrated in order to achieve recalibration. In the present study, we investigated whether recalibration due to the McGurk stimulus requires the incongruent audiovisual inputs to be fused (e.g. McGurk illusion) or whether exposure to the audiovisual conflict is sufficient to elicit recalibration. We hypothesized that recalibration may only occur when the McGurk illusion was perceived, thus if auditory and visual inputs were fused to ‘ada’. In keeping with a Bayesian belief-updating model [7], we reasoned that experiencing the McGurk illusion leads to a shift in the input–percept–mapping, i.e. an /aba/ sound (*input*) is categorized as ‘ada’ (*percept*) and consequently, the mapping between input and percept is updated (i.e. recalibration). This shift should transfer to /aba/ stimuli categorization on the subsequent trial. When participants do not experience the McGurk illusion, there is no such shift and recalibration may not occur.

Secondly, it is unclear whether and how phonetic recalibration depends on sensory uncertainty. Perception results from an optimal (or near-optimal) combination of different sensory inputs [8,9]. The weight of each sensory input in the final percept is determined by its relative sensory uncertainty. When one sensory input is noisier compared to other sources of information (e.g. close past events or other sensory modalities), its weight in determining the final percept is reduced [10,11]. Thus, we predicted that an increase in the uncertainty of the auditory input due to increments in sensory noise should lead to a corresponding increase in the relative weight of the percept on the preceding trial. Based on this prediction, we expected the strongest recalibration if the current auditory input was noisy.

**Figure 1.**
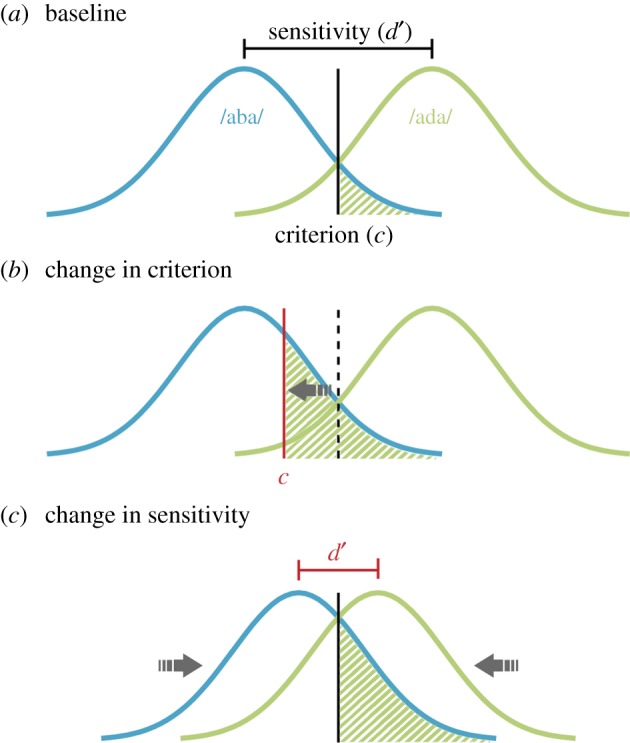
Possible mechanisms underlying the recalibration effect after a McGurk illusion. (*a*) In a signal detection framework, two slightly overlapping curves represent the phonemes /aba/ (blue, left) and /ada/ (green, right). The distance between the two curves depicts the sensitivity *d*′. The criterion (vertical line) determines the perceptual label. Everything that falls to the left of the criterion is categorized as ‘aba’ and everything on the right as ‘ada’. The green shaded area depicts the false alarms; in other words, the cases when /aba/ is misperceived as ‘ada’. (*b*) A shift in the criterion leads to an increased percentage of misperceived /aba/, i.e. a larger shaded area compared to (*a*). This mechanism would be expressed in a decreased criterion after a McGurk illusion. (*c*) Shifting the representations closer to each other could also account for an increased percentage of misperceived /aba/. This mechanism would be expressed in a decreased sensitivity *d*′ after a McGurk illusion.

Thirdly, it is unclear whether phonetic recalibration is the result of a bias or a shift in phonetic representation. Recalibration could be caused by two mechanisms (figure 1). From a signal detection theory (SDT) perspective [12], a change in criterion (generally respond ‘ada’ more often; figure 1*b*), a change in sensitivity (/aba/ and /ada/ become more similar; figure 1*c*) or a combination of both can all lead to an increase in ‘ada’ percepts for /aba/ sounds. While a change in criterion can correspond to decisional or a perceptual bias, a change in sensitivity would suggest a purely perceptual phenomenon [13]. We predicted that if phonetic recalibration results in the phoneme /aba/ becoming perceptually more similar to /ada/, this should be reflected in a decreased sensitivity to discriminate /aba/ and /ada/ stimuli after a McGurk illusion. On the other hand, if phonetic recalibration simply involves a change in the phonetic boundaries between /aba/ and /ada/ without a change in the sensory representation, we do not expect to find a change in the sensitivity, but only a criterion shift [13].

To preview, we found a strong recalibration effect specifically when the McGurk illusion was perceived on the previous trial. This recalibration effect was associated with a change in both criterion and discrimination sensitivity. Furthermore, the sensory similarity between the stimuli on two consecutive trials determined whether recalibration took place, suggesting that audiovisual recalibration is a stimulus-specific effect.

## 2. Methods

### 2.1. Participants

Fifty-four participants (of which 38 were women) took part in this behavioural study. We used a large sample to allow for broad variability in the strength of the McGurk illusion. The majority was Dutch (22) or German (17) and all understood the English instructions. They had normal or corrected-to-normal vision, normal hearing and gave written informed consent in accordance with the institutional guidelines of the local ethical committee (CMO region Arnhem-Nijmegen, The Netherlands) and were either financially compensated or received study credits for their participation.

### 2.2. Stimuli

**Figure 2.**
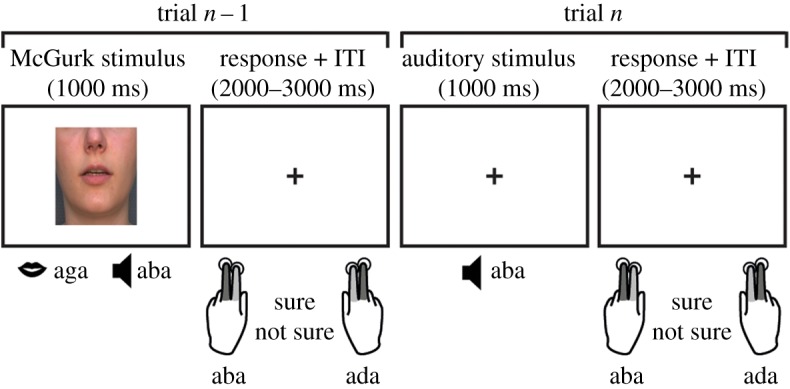
Experimental design. An audiovisual or auditory stimulus was presented on the screen for 1 s followed by a fixation cross-period of 1500 ms in which participants pressed a button to indicate whether they heard ‘aba’ or ‘ada’. When they were not sure, they pressed with their index finger and when they were sure, they pressed with their middle finger. The next trial appeared after a jitter of 500–1000 ms.

Audiovisual and auditory speech stimuli were presented to the participants on a 24-inch screen (60 Hz refresh rate) and two speakers located under the screen. The audiovisual stimuli showed the lower part of the face of a speaker uttering syllables (figure 2). They were presented centrally and covered 6% of the screen (visual angle approx. 8°). A female speaker was recorded with a digital video camera in a soundproof room while uttering /aba/, /ada/ and /aga/. The videos were edited in Adobe Premier Pro CS6 such that the mouth was always located in the centre of the screen to avoid eye movements between trials. After editing, each video started and ended with a neutral mouth slightly opened such that participants could not distinguish the videos based on the beginning of the video. All videos were 1000 ms long with a total sound duration of 720 ms. Only the lower part of the face from the nose to chin was visible in the videos to prevent participants' attention being drawn away from the mouth to the eyes. All syllables were presented at approximately 60 dB. The McGurk stimuli trials (31% of the total trials) were created by overlaying /aga/ movies to the sound of an /aba/ video. To answer our question, we were mainly interested in analysing McGurk, auditory /aba/ and /ada/ trials. However, only including audiovisual incongruent trials can lead to fewer illusions [14]. We therefore also included a few audiovisual congruent /aba/ and /ada/ trials (8% of the total trials). In total, there were nine audiovisual videos, three for every condition (audiovisual /aba/, audiovisual /ada/, McGurk). In contrast with the audiovisual trials, during the auditory-only trials (61% of the total trials), /aba/ or /ada/ was played while participants fixated at a grey cross in the centre of the screen where the mouth appeared during the audiovisual trials. To investigate whether sensory uncertainty interacts with phonetic recalibration, we added white noise in the background of the sound files on half of the audiovisual and auditory-only trials. For this purpose, any noise that was inherent to the recordings were first removed with the software Audacity (www.audacityteam.org). Afterwards, white noise with two 100 ms ramps (to prevent sound bursts) at the beginning and end of the stimulus was overlaid onto the sound files. The volume of the noise was determined for each participant before the start of the experiment using a one-up-one-down staircasing procedure (30 reversals) while participants had to categorize auditory /aba/ and /ada/. On average, the noise was 10 dB louder than the syllable. All the stimuli were presented using the Presentation software (www.neurobs.com).

### 2.3. Procedure

On each trial, audiovisual and auditory stimuli were presented for 1 s. Participants had 2–3 s after each stimulus before a new stimulus appeared to report what they had heard. They were, however, instructed to always focus and attend to the mouth. When the fixation cross appeared on the screen, they indicated with a single button press what they had heard and how confident they were about it (figure 2). They responded with their left hand for /aba/ and with their right hand for /ada/. If they were confident about their percept, they were instructed to use their middle finger and if they were not confident their index finger (see light and dark grey marked fingers in figure 2). In between stimuli, a grey fixation cross was displayed that was also present during the auditory trials to minimize eye movements.

Since we were interested in trial order effects, we created stimulus sequences in Matlab, such that the conditions of interest (McGurk, /aba/, /ada/) followed each other equally often. Many random permutations of trial orders were created until the sequence fulfilled our criteria, i.e. about 25 trial pairs per condition (e.g. noisy /aba/ preceded by non-noisy McGurk). To make sure that the results do not depend on a specific trial order, we created five different trial orders that fulfilled the criteria and counterbalanced those across participants. The number of trials per condition of interest was 158 (McGurk, /aba/ and /ada/) and 20 for the filler items (congruent /aba/ and /ada/) yielding 1028 trials in total. On average, this yielded 24 trials per condition pair of previous and current trial (see electronic supplementary material, table S2 for all trial orders). The experiment took place in cubicle laboratories where up to four participants were tested in parallel. Sounds from neighbouring cubicles were inaudible to the participants. The experiment was split into eight blocks of approximately 8 min. During the breaks, participant rested for at least half a minute. Every second break, they were asked to open the door and briefly interacted with the researcher to monitor participants’ well-being.

### 2.4. Analysis

Our experimental design consisted of auditory and audiovisual speech trials, which could be either embedded in white noise or not. All responses by the participants were categorized into ‘aba’ and ‘ada’. We were interested in the effect of the previous trial on the percept on the current trial. Therefore, we looked at the percept on auditory /aba/ and /ada/ trials that were preceded by either /aba/, /ada/ or McGurk illusion (‘ada’ percept). Only when addressing the question whether it is necessary for recalibration to fuse McGurk stimuli, we split McGurk trials into fused (‘ada’ percept) and non-fused trials (‘aba’ percept). For all remaining questions, we restricted the analysis to fused McGurk trials. Since the influence of the perceptual interpretation on the next trial was crucial in our experiment, we only included preceding /aba/ and /ada/ trials that were correctly perceived (e.g. /aba/ preceded by /ada/ which was correctly perceived as ‘ada’). To have as many trials per condition as possible, we combined trials with high and low confidence ratings. The confidence ratings were only analysed for misperceived /aba/ and /ada/ trials to investigate how certain participants were even if they were wrong, and specifically whether they were more certain about their (wrong) percept after a McGurk illusion. We addressed this question with the binary confidence ratings that were given on every trial (see Procedure and figure 2). We compared the percentage of high confidence ratings during misperceived /aba/ and /ada/ trials after a McGurk illusion with those after correct /ada/.

To investigate whether recalibration was reflected in a shifted criterion or a decreased sensitivity to discriminate /aba/ from /ada/, we applied SDT to the data. For this analysis, many trials were necessary to achieve stable estimates for *d*′ and the criterion. We therefore combined low and high noise trials and excluded data points with less than 10 trials. All 54 participants were included in this analysis. We looked at /ada/ and /aba/ trials that were perceived as ‘ada’ (hits and false alarms, respectively) or ‘aba’ (misses and correct rejections, respectively). The sensitivity *d*′ (*z*(hit)−*z*(FA)) and the criterion *c* ($-\frac{1}{2}*$(*z*(hit) + *z*(FA)) were computed for all trials preceded by /aba/, /ada/ and a McGurk illusion. Since extreme performance of 0% and 100% lead to infinite values for *d*′, we adjusted these proportions to 1/2*N* and 1 − 1/2*N*, respectively, where *N* is the number of trials [15]. We compared the sensitivity *d*′ (dependent variable) for all trials after /aba/, /ada/ and McGurk illusions (within-subject factor). Additionally, two post hoc paired *t*-tests were performed to see whether the sensitivity after a McGurk illusion was different from after /ada/ and /aba/. The same steps were performed for the criterion. Additionally, we compared *d*′ and the criterion after fused and non-fused McGurk trials in two paired *t*-tests. Since the overall proportion of McGurk illusions was high (72%) and because we restricted the SDT analysis to participants with at least 10 trials, only 31 participants with a sufficient number of fused and non-fused McGurk trials could be included in this analysis.

## 3. Results

### 3.1. Phonetic recalibration after McGurk illusion

**Figure 3.**
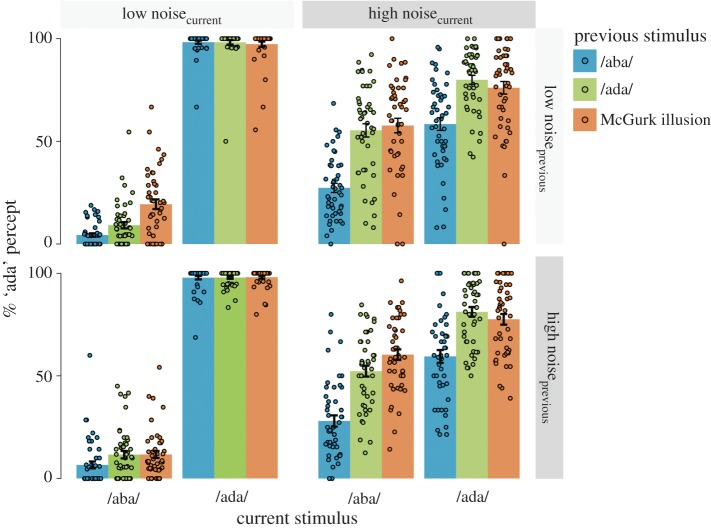
Responses on all conditions. The percentage of ‘ada’ responses during auditory /aba/ and /ada/ trials are shown here (*N* = 47). The colours of the bars indicate the condition of the previous trial: /aba/ (blue), /ada/ (green) and McGurk illusion (auditory /aba/ and visual /aga/, orange). The dots display individual subjects. Only previous correct trials and fused McGurk trials (perceived as ‘ada’) were included. The left two quadrants depict trials without added acoustic noise and the right ones depict trials with acoustic noise. The lower two quadrants show trials that were preceded by noisy trials. The recalibration effect (more ‘ada’ percepts during /aba/ after McGurk) was strongest if the previous and current trial had the same noise level, i.e. upper left and lower right quadrants. This is reflected in a larger percentage of ‘ada’ during /aba/ after McGurk (orange bar) than after /ada/ (green bar). Error bars display the standard errors of the mean.

**Figure 4.**
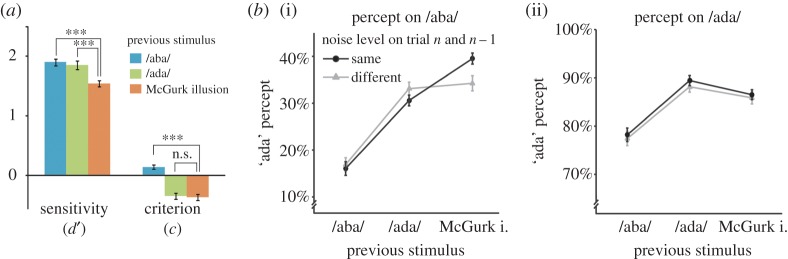
Recalibration effect after McGurk illusion. (*a*) After a fused McGurk stimulus, the sensitivity (*d*′) was on average smaller (orange) than after auditory /aba/ (blue) or /ada/ (green). The criterion shifted away from /ada/ after McGurk as well as after /ada/ trials implying a bias towards the previous percept ‘ada’. *N* = 54. (***p < 0.001; n.s., non-significant). (*b*) Recalibration is strongest for the same noise level. The percentage of ‘ada’ percepts during auditory /aba/ (i) and /ada/ trials (ii) are shown. When the noise level on the previous and current trial was the same (both low or high, black circles), participants often misperceived /aba/ as ‘ada’ after a McGurk illusion (McGurk i.). If the noise was only high on the previous or current trial (grey triangles), the effect was weaker. This figure illustrates the four-way interaction between current condition × previous condition × current noise × previous noise. Error bars display within-subjects standard errors.

In a previous study [3], we observed an increase in /aba/ stimuli being perceived as /ada/ after a McGurk illusion. To investigate whether we could replicate this recalibration effect, we ran a repeated-measures analysis of variance (ANOVA) with condition and noise on the previous and current trial as within-subjects factors: (i) condition on the current trial (McGurk, auditory /aba/ and /ada/), (ii) condition on the previous trial (McGurk, auditory /aba/ and /ada/), (iii) acoustic noise on the current trial (low, high), and (iv) acoustic noise on the previous trial (low, high) (figure 3 for the data on all conditions). Some participants did not experience the McGurk illusion very often on the non-noisy trials. As a consequence, it happened that in some participants, some conditions were never preceded by a non-noisy McGurk illusion. Thus, only 47 of the 54 participants could be included in the analysis. We looked at the percentage of ‘ada’ percepts as dependent variable. We predicted an interaction between the condition on the previous and current trial in the percentage of ‘ada’ percepts. In line with the previous study, we restricted this analysis to McGurk illusions (fused to ‘ada’). Indeed, perception was dependent on both the current and previous stimulus (current × previous stimulus: *F*_2,92_ = 26.86; *p* = 2.1 × 10^−8^, $\eta_{\text{partial}}^{2}=0.54$). Specifically, the perception of /aba/ stimuli was more biased towards /ada/ after a McGurk illusion than after an /ada/ sound (37.5% versus 31.9%: *t*_46_ = −4.53, *p* = 4.2×10^−5^), whereas for perception of /ada/ sounds, this pattern reversed, with more ‘ada’ percepts after /ada/ (86.8 versus 88.7%: *t*_46_ = 2.13, *p* = 0.038). In other words, when the previous trial was perceived as ‘ada’, this led to an increase in misperceived /aba/ trials but more so after a McGurk illusion than after uni-sensory /ada/ (figure 4*b*). This replicates our previous finding that perceiving the McGurk illusion recalibrates how /aba/ is perceived [3].

To characterize the perceptual nature of the phonetic recalibration effect, we analysed the data within an SDT framework. Interestingly, we found that sensitivity (*d*′) differed significantly for trials that were preceded by either /aba/, /ada/ or a McGurk illusion (*F*_2,106_ = 22.78, *p* = 7.9 × 10^−8^). Specifically, *d*′ was smaller after a McGurk illusion (*M* = 1.58, s.d. = 0.41) than after /aba/ (*M* = 1.94, s.d. = 0.46, *t*_53_ = 6.49, *p* = 3.0 × 10^−8^) or /ada/ (*M* = 1.89, s.d. = 0.51, *t*_53_ = 5.02, *p* = 6.2 × 10^−6^, figure 4*a*). This decrease in sensitivity indicates that /aba/ and /ada/ were perceived more similarly after a McGurk illusion. Apart from a change in sensitivity, we also observed a significant effect of the previous trial on the criterion (*F*_2,52_ = 58.93, *p* = 4.3 × 10^−14^, figure 4*a*). Subjects were more biased towards ‘ada’ following either a McGurk illusion (*C* = −0.40, s.d. = 0.35) or an /ada/ trial (*C* = −0.37, s.d. = 0.36), compared to following an /aba/ trial (*C* = 0.15, s.d. = 0.33). Post hoc tests on the criterion parameter showed that the participants were equally biased to report ‘ada’ following McGurk illusions and /ada/ trials (*t*_53_ = 0.87, *p* = 0.39).

The previous analysis showed that McGurk illusion decreases discrimination sensitivity on subsequent trials. Next, we examined whether this also decreased participants' confidence. The same 47 subjects were included as in the replication analysis. However, two participants always correctly perceived /ada/ and could therefore not be included. The repeated-measures ANOVA was performed on the remaining 45 participants. Interestingly, participants were more confident about their misperceived /aba/ stimulus after a McGurk illusion (35.8% high confidence) than after an /ada/ trial (31.2%). This pattern was reversed for misperceived /ada/ trials where they were more confident after /ada/ than after McGurk (25.2 versus 21.1%; current × previous stimulus: *F*_1,44_ = 4.32; *p* = 0.043). In other words, both, a McGurk illusion and auditory /ada/ biased participants towards ‘ada’ on the following trial, but the decreased sensitivity and high confidence ratings support the idea that /aba/ is truly perceived differently after a McGurk illusion and that participants are not merely biased by their preceding percept. While there is a general bias (i.e. criterion) induced by the previous stimulus (which is also present after /ada/ and /aba/ trials), the McGurk illusion also pulls the perception of /aba/ sounds towards /ada/, thereby making these stimuli less discriminable and reducing perceptual sensitivity (*d*′).

### 3.2. Recalibration depends on perceiving the McGurk illusion

**Figure 5.**
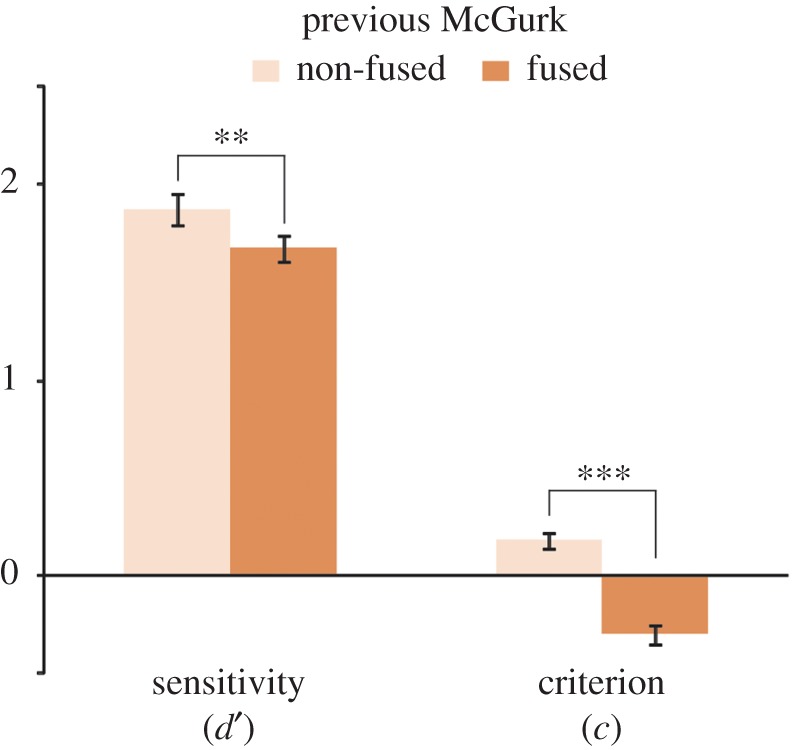
Recalibration depends on perceiving the McGurk illusion. McGurk trials were split into non-fused (‘aba’ percept, light orange) and fused (‘ada’ percept, dark orange) trials. When participants fused the preceding McGurk stimulus, the sensitivity *d*′ to distinguish /aba/ from /ada/ was smaller compared to when they did not fuse it. After a McGurk illusion, participants gave more ‘ada’ responses (negative criterion shift) while after a non-fused McGurk stimulus, they responded ‘aba’ more often (positive criterion shift). *N* = 31. (**p < 0.01; ***p < 0.001).

To investigate whether it is necessary for the recalibration effect that the McGurk illusion was perceived on the previous trial, we split McGurk trials into fused (‘ada’ percept) and non-fused trials (‘aba’ percept). Five participants perceived the McGurk illusion on all trials and could therefore not be included in this analysis (*N* = 49). After a McGurk illusion, auditory /aba/ was more often perceived as ‘ada’ (35.1%) than after McGurk trials that were not fused (21.2%; *t*_48_ = 3.93, *p* = 2.7 × 10^−4^). Thus, recalibration was markedly stronger after fused McGurk trials (illusion) than after non-fused McGurk trials (no illusion). This comparison leaves the possibility that there still was some recalibration effect after non-fused McGurk trials, which simply was weaker than after a McGurk illusion. Since recalibration should lead to a larger amount of misperceived /aba/ trials than by mere priming of an ‘ada’ percept [3], we compared perception of /aba/ trials after non-fused and fused McGurk trials to those after auditory /ada/. While /aba/ was more often misperceived after a McGurk illusion than after auditory /ada/ (35.1% versus 29.8%, *t*_48_ = 4.28, *p* = 8.8 × 10^−5^), participants were less likely to misperceive /aba/ as ‘ada’ after a non-fused McGurk trial (21.2%) than after an /ada/ trial (29.8%, *t*_48_ = 2.26, *p* = 0.028). This suggests that only fused McGurk trials elicited a recalibration effect. As a complementary analysis, we again looked at the sensitivity and the criterion after a McGurk trial, separately for fused and non-fused McGurk trials. Immediately after a fused McGurk, the sensitivity (*d*′) was smaller (*M* = 1.67, s.d. = 0.38) than after a non-fused McGurk trial (*M* = 1.88, s.d. = 0.46; *t*_30 _= −3.48, *p* = 0.002, figure 5). *d*′ after a non-fused McGurk was not smaller than after /ada/ (*t*_30_ = 0.65, *p* = 0.52), again suggesting that there was no recalibration effect after non-fused McGurk stimuli. In other words, /aba/ and /ada/ were more difficult to distinguish if the previous McGurk trial was fused to ‘ada’ compared to when it was not fused. The criterion to report ‘ada’ after a fused McGurk (*M* = −0.31, s.d. = 0.30) was more liberal (more ‘ada’ responses), while after a non-fused McGurk, it was more conservative (less ‘ada’ responses; *M* = 0.18, s.d. = 0.25; fused versus non-fused: *F*_1,30_ = 85.26, *p* = 2.83 × 10^−10^, figure 5). This suggests that a McGurk trial biased participants to respond ‘ada’ on the next trial but only if it was fused to ‘ada’. These results suggest that the perceptual interpretation of McGurk stimuli rather than the exposure to audiovisual conflict determines the effect on the next trial, namely how /aba/ is perceived.

### 3.3. Recalibration effect does not generalize between sounds

To manipulate sensory uncertainty, auditory stimuli could be embedded within white noise. Categorizing the syllables /aba/ and /ada/ in the presence of white noise was more difficult for noisy stimuli (accuracy for /aba/: 55.2%, /ada/: 69.6%) than for stimuli with no noise (accuracy for /aba/: 90.0%; /ada/: 97.2%; main effect of noise: *F*_1,53_ = 619.36, *p* = 6.6 × 10^−31^). Moreover, the proportion of ‘ada’ and ‘aba’ responses was more biased in the direction of the percept on the previous trial in the high noise conditions. These results demonstrate that our acoustic noise manipulation effectively increased sensory uncertainty, showing a decreased performance in phoneme discrimination and increments in perceptual priming under high noise stimulation [16]. To investigate the effect of noise on McGurk illusions, we ran a separate analysis (paired *t*-test) where we looked at the number of illusions with or without noise. We observed that 71.8% (± 26.6%, mean ± s.d.) of McGurk trials were fused to ‘ada’. Participants were more likely to fuse McGurk stimuli when the auditory input contained noise (*M* = 84.4%, s.d. = 18.6%) than when it did not contain noise (*M* = 59.2%, s.d. = 38.0%, *t*_53_ = 6.74, *p* = 1.2 × 10^−8^), suggesting that their perceptual judgement was more influenced by the visual input if the auditory input was noisy.

Next, we addressed the question whether the recalibration effect was modulated by uncertainty in the auditory domain. Owing to absent trials preceded by non-noisy McGurk illusions in seven subjects (see §3.1), again this analysis was carried out on 47 participants. We expected that the recalibration effect was also affected by noise, in other words, a stronger influence of the previous McGurk illusion trial on the current /aba/ trial if the current trial was noisy. However, we did not find a modulation of the recalibration effect by noise which would be reflected in a three-way interaction between noise, previous condition and current condition. Neither noise on the current (*F*_1,46_ = 0.97, *p* = 0.39) nor on the previous trial (*F*_1,46_ = 0.76, *p* = 0.47) modulated the recalibration effect. Instead, we found a significant four-way interaction for the percentage of ‘ada’ percepts between condition and noise level on the previous and current trial (*F*_2,45_ = 4.12, *p* = 0.023). Unpacking this complex interaction, we observed that recalibration after McGurk illusions occurred specifically for /aba/ trials (and not /ada/ trials), and only when the noise level (and therefore the sound) of the current trial was identical to the noise level of the previous trial (recalibration for identical noise levels: 9.0% (39 − 30%) more ‘ada’ after McGurk illusion than after /ada/; recalibration for different noise levels: only 1.1% (34 − 33%) more ‘ada’; figure 4*b*). Interestingly, there was only a significant interaction between the noise level on the previous and current trial for auditory /aba/ trials that were preceded by a McGurk illusion (*F*_1,46_ = 7.01, *p* = 0.011). For trials with low noise, on average, 19.2% of /aba/ trials were perceived as ‘ada’ after a McGurk illusion trial. However, if the McGurk trial contained a different noise level (i.e. high noise), this was only 11.6%. The percentage of misheard /aba/ trials in the high noise condition was overall larger, but the same reasoning applied for trials with high noise, i.e. more recalibration if the previous and current trial included white noise. About 59.9% of the /aba/ trials were perceived as ‘ada’ after a fused McGurk trial. However, if the McGurk trial contained low noise, this was only 56.9%; see figure 3 for the data on all conditions). We did not find such an interaction effect for any other conditions—/aba/ preceded by /ada/ (*F*_1,46_ = 3.19, *p* = 0.081), /ada/ preceded by McGurk (*F*_1,46_ = 0.16, *p* = 0.69) or /ada/ preceded by /ada/ (*F*_1,46_ = 0.93, *p* = 0.34). Thus, we found the strongest recalibration effect for trials with the same level of auditory noise.

## 4. Discussion

**Figure 6.**
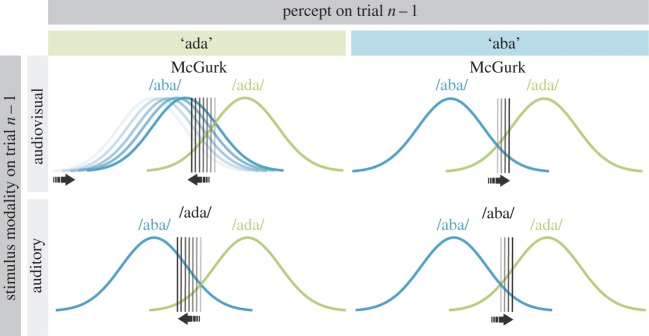
Schematic of the observed phonetic recalibration. The two curves display the perceptual representations of /aba/ (blue) and /ada/ phonemes (green). Depending on the preceding trial (*n *− 1) /aba/ and /ada/ might be distinguished using a different boundary (criterion shift) or move closer to each other (decreased sensitivity). After a McGurk illusion (auditory /aba/ together with an /aga/ video was perceived as ‘ada’; upper left quadrant), not only did the criterion shift towards /aba/ (black vertical line) but the two curves also moved closer to each other making /aba/ and /ada/ less distinguishable. If the preceding McGurk stimulus, however, was not fused (perceived as ‘aba’; upper right quadrant), then only a shift in criterion in the opposite direction was observed. In that respect, the effect of a McGurk trial perceived as ‘aba’ on the consecutive trial was comparable to that of auditory /aba/ (lower right quadrant) where we see a criterion shift in the same direction. After an auditory /ada/, the criterion shift was comparable to that after a McGurk illusion but without a different sensitivity. (For the numeric results of the SDT analysis, see figures 4 and 5.)

In this study, we addressed the mechanisms and requirements of phonetic recalibration. We studied a large sample of participants with varying strengths of an audiovisual speech illusion (McGurk illusion: auditory /aba/ together with visual /aga/ perceived as ‘ada’). This enabled us to look at the relevance of integrating audiovisual information in order to induce phonetic recalibration effects. We found that integrating audiovisual information is necessary to undergo phonetic recalibration. Specifically, we found that immediately after experiencing the McGurk illusion, but less after failing to perceive the illusion (21%), auditory /aba/ was regularly misperceived as ‘ada’ (35%). SDT-based analyses showed that this recalibration involved a perceptual shift. Auditory /aba/ became perceptually more similar to /ada/ after a McGurk illusion, which was evident from a decreased sensitivity. Furthermore, we observed that the similarity (in the level of acoustic noise) between the stimuli in two consecutive trials determined the size of the recalibration effect. The more similar they were, the stronger the effect was. Below we will describe and interpret the results in more detail (figure 6 for a schematic display of the main findings).

### 4.1. Recalibration depends on audiovisual integration

It is generally thought that ‘recalibration occurs only when there is a conflict between auditory speech and visual speech’ [6]. However, up to now, it was unclear whether exposure to audiovisual conflict is sufficient to elicit phonetic recalibration or whether audiovisual inputs have to be integrated to one percept. This stems partly from the fact that the percept at the moment of the audiovisual conflict is unknown. Phonetic recalibration is typically studied using an exposure to audiovisual conflicting information and a phonetic categorization task [1,4]. During the exposure, phase participants are usually not asked what they perceive. It is therefore unknown whether they successfully integrated the audiovisual information on all trials. Ceiling performance during an identification task of the audiovisual stimuli after exposure suggests that an ambiguous sound was indeed interpreted in line with the visual speech. So, indeed, it seems like audiovisual information was successfully integrated. However, the question remained whether it is necessary for recalibration to integrate conflicting inputs or whether exposure to conflict is sufficient. In this paper, we have asked participants on a trial-by-trial basis to report how they perceived the incongruent McGurk stimulus (auditory /aba/ and visual aga/ integrated to ‘ada’ or not). We have found that it is indeed necessary that a McGurk stimulus is fused to ‘ada’ to elicit phonetic recalibration. If it is fused, then the next /aba/ sound that is encountered is more likely to be perceived as ‘ada’ than after mere auditory /ada/. Crucially, when the McGurk illusion was not perceived (‘aba’ percept), the following /aba/ stimulus was perceived as ‘aba’ more often. While there was a general decision bias towards the percept on the previous trial (reflected in a more liberal criterion to report ‘ada’ following /ada/ and McGurk trials), only after a fused McGurk, we found decreased sensitivity, indicative of perceptual recalibration. The sensitivity (*d*′) to distinguish /aba/ from /ada/ was smaller after a McGurk illusion than after no illusion. The fact that we only found an effect on the sensitivity after a McGurk illusion gives further support that our finding reflects more than mere priming and rather a perceptual shift [3]. In line with our results, van Linden & Vroomen [17] showed that the strength of the recalibration after-effect is highly determined by the percept during adaptor. Specifically, they showed that the size of the bias induced by lexical context in a phoneme categorization task was positively correlated with the strength of the induced after-effect. Here, we go further by showing that exposure to audiovisual conflict is not sufficient to elicit phonetic recalibration. Instead, audiovisual inputs have to be integrated to one percept to elicit phonetic recalibration. It is in line with studies using sinewave speech (i.e. artificially altered speech that is not perceived as speech by naive listeners) where phonetic recalibration only takes place if the auditory information is interpreted as speech [4] and as a prerequisite is integrated with the lip movements of a speaker [18]. Together with our findings, it suggests that the perceptual interpretation of audiovisual events have impact on the subsequent uni-sensory percept.

Recalibration can occur after audiovisual integration in different dimensions—in time [19], space [20] and identity [1]. The current study focused on the last of these, the identity of speech sounds. We demonstrated rapid recalibration, a shift in perception after a single exposure to the McGurk illusion. It is an example of the flexibility of the brain, for instance, to quickly adjust to different speaker characteristics. Rapid recalibration that does not require long exposure time has previously been demonstrated in the temporal [21] as well as in the spatial domain [20] and for the categorization of phonemes [22]. In audiovisual temporal recalibration, conflicting auditory and visual information in time is perceived as synchronous after brief exposure to the asynchrony [19]. Contrary to our findings, in the temporary domain, recalibration can occur independent of the percept, i.e. independently of whether the preceding asynchrony was perceived as such [21,23]. One potential explanation for this difference might be that temporal integration of audiovisual events occurs before speech integration. Temporal integration is a prerequisite for the McGurk effect—only if auditory and visual speech occur close enough in time, then there can be integration that leads to the McGurk illusion [24,25]. It might therefore be more automatic and require less awareness than the interpretation of speech. Even if participants perceive the speech as asynchronous, the brain might make use of the information outside the awareness of the participants. The integration of audiovisual speech, however, requires cognitive control [26,27].

### 4.2. Recalibration does not generalize between sounds

We expected that preceding McGurk illusions lead to a stronger recalibration effect if the auditory estimate is uncertain. Somewhat surprisingly, we did not find stronger recalibration in the uncertain condition (i.e. added white noise). While multi-sensory integration generally depends on the reliabilities of the sensory information [28], recalibration does not seem to depend on how reliable the uni-sensory inputs are. Instead of an effect of uncertainty, we found the strongest recalibration effect if the previous and the current trial share most acoustic features, i.e. if the noise level was the same. It suggests that recalibration is very specific to the encountered sound.

Previous research does not agree on whether recalibration generalizes across speakers [29–31]. Our results support the idea that phonetic recalibration is very sensitive to the similarity and interpretation of previous stimulation. Based on our findings, we would predict that phonetic recalibration does not generalize across speakers because the spoken syllables from different speakers would differ in their acoustic features and therefore not elicit recalibration. This view is compatible with previous studies showing a speaker-specific effect [29,31]. Interestingly, the recalibration effect which shifts the percept of the phoneme /b/towards /d/ does not generalize from /aba/ to slightly different syllables like /ubu/ or /ibi/ [32]. In other words, even if the phoneme is exactly the same /b/ during exposure and testing, the acoustic features during exposure (in this case, the surrounding phonemes) determine whether recalibration occurs. In our case, also the same /b/ was encountered, but the acoustic context (noise) was sometimes different. Phonetic recalibration after-effects have been found to not only be phoneme- or speaker-specific, but also ear-specific. Keetels *et al*. [33] showed that the same ambiguous sound can be simultaneously adapted to two opposing phonemic interpretations if presented in the left and right ear. All these results together suggest that phonetic recalibration is very specific to the acoustic context during exposure. Such stimulus specificity might be adaptive to update the phonetic mappings of different speakers in parallel. This mechanism seems plausible because similar phonemes can produce quite different acoustic features. Phonetic recalibration should take these variations into account and therefore be a very speaker-specific mechanism. How the brain updates and stores these multiple input–percept mappings in parallel remains a question for future investigations.

### 4.3. Phonetic recalibration makes /aba/ more similar to /ada/

With the current study, we aimed at unveiling the perceptual and decisional nature of phonetic recalibration. To do so, we used the SDT to index changes in sensitivity and criterion. As described earlier (see Introduction and figure 1), an increase in ‘ada’ responses during auditory /aba/ could be caused by a shifted criterion (more ‘ada’ responses) or a perceptual change in sensitivity (/aba/ and /ada/ become more similar). We applied SDT and found support for both (figure 4*a* for the numeric results; figure 6 for a schematic illustration of the results). After experiencing a McGurk illusion, the criterion was more shifted towards /aba/ than after experiencing a non-fused McGurk or an ‘aba’ trial, and thereby increased the proportion of ‘ada’ responses. This was also found after auditory /ada/ trials (figure 6 lower two panels). The criterion shift can therefore be seen as a form of perceptual priming by the previous trial. More interestingly, we found a change in sensitivity only after McGurk illusion trials. In other words, after participants fused auditory /aba/ and visual /aga/ to ‘ada’, then auditory /aba/ and /ada/ became more similar. Solely based on the decreased sensitivity, we cannot tell whether /aba/ becomes more similar to /ada/, /ada/ more like /aba/ or both. However, because auditory /ada/ is affected less by a preceding McGurk illusion than /aba/, it seems likely that /aba/ shifted towards /ada/ and not the other way around. In other words, after experiencing the McGurk illusion, auditory /aba/ actually sounds a bit more like ‘ada’ which was the fused percept on the trial before (figure 6 upper left panel).

### 4.4. Support for a Bayesian belief-updating model

Our recalibration results, in particular the shifted distribution of /aba/ towards /ada/ after a McGurk illusion, can be accounted for by a Bayesian belief-updating model [7]. According to this model, when listeners encounter a sound (e.g. /b/ during McGurk stimulus) that they consider to be /d/ (e.g. McGurk illusion), they will change their beliefs about the distribution of /b/. The simulations by Kleinschmidt & Jaeger predicted a shift of /aba/ towards /ada/, which is exactly what we found. It also explains why there is and should be no recalibration after a non-fused McGurk trial. Namely, when listeners do not consider /b/ to be /d/, there is no update of the belief-model. The updated model should continue incorporating new evidence. We therefore speculate on the persistence of the recalibration effect on adjacent trials. Two consecutively misperceived /aba/ sounds (during McGurk and recalibrated /aba/) should strengthen the updated belief (shift towards /d/). However, new counter-evidence (/aba/ perceived as ‘aba’) after a McGurk illusion should weaken this belief (shift towards /b/). In an exploratory analysis (see electronic supplementary material), we tested this hypothesis. We compared /aba/ trials after McGurk-induced recalibration with /aba/ trials after correctly perceived /aba/. We observed that even two trials after a McGurk illusion, the probability that /aba/ was misperceived as ‘ada’ was larger after a misperceived /aba/ than after correct /aba/. This result provides additional support for the belief-updating model. If new incoming evidence after a McGurk illusion is veridically interpreted (i.e. /aba/ stimulus = ‘aba’ percept), the prior /b/ distribution is shifted less towards the /d/ distribution than after a recalibrated /aba/ that was perceived as ‘ada’.

As far as we know, this is the first empirical data on phonetic recalibration caused by audiovisual speech that supports the proposed Bayesian belief-updating model. Besides visual speech, phonetic recalibration can be triggered by a lexical context, e.g. a word [34]. An ambiguous sound in between two phonemes (e.g. between /f/ and /s/) is perceived as ‘s’, if it occurred before in an s-word. For this type of recalibration, a similar discovery was made. Recalibration due to lexical context was reflected in a shifted criterion as well as in a decreased sensitivity [35]. A consistent finding in the literature is that lip-read recalibration is usually stronger than lexical recalibration [17]. This might appear counterintuitive from a Bayesian belief-updating model, given that lexical context is more constraining than lip-read context (e.g. a lip closure may signal either /b/, /p/ or /m/). This may lead to the *a priori* prediction that the less ambiguous lexical context should update the observer's belief more strongly than lip-read context, leading to stronger recalibration after-effects. We speculate that a possible explanation for this asymmetry in recalibration strength might be related to the degree of estimated causal link for conflicting information derived from lip-read and lexical contexts. We propose that integrating auditory speech and lip-read context (McGurk illusion) involves the assumption of a more direct causal link between both sources of information than the integration of higher-level phonetic representations (lexical recalibration). Unlike lexical context, lip-read and auditory phonetic stimuli generally hold very consistent spatio-temporal correlations (i.e. auditory speech signals and lip-read signals unfold over time and contain both dynamic configural information and local motion cues). That means that conflicts between lip-read and auditory signals can be resolved at the stimulus level, reinforcing the unity assumption between the two external sources of phonetic information. Assuming a stronger causal link between lip-read and auditory information can explain why a listener updates its /aba/ distribution more often in the case of lip-read recalibration paradigms, leading to stronger recalibration effects compared to lexical recalibration paradigms.

Together with the current study, a Bayesian belief-updating model supports the idea that categorization of phonemes can shift perceptually to account for variabilities in the environment. Our finding that the recalibration effect seems stimulus-specific points to the possible nature of these variabilities. Namely, that the brain can adjust to specific variabilities but that it does not generalize easily. This intuitively makes sense because we learn to categorize continuous sounds into discrete phonemes and words. A strong prior about the phoneme boundaries is likely to have been built throughout our life. If a counter-example is encountered (e.g. a speaker pronouncing a phoneme slightly different), it seems reasonable to adjust the interpretation of that specific sound and not the whole category.

### 4.5. Selective speech adaptation versus phonetic recalibration

Our study replicates previous findings showing that conflicting visual context induces strong speech recalibration after-effects [1,3,36]. However, it is important to note that we did not find selective speech adaptation, which is another type of after-effect [37]: hearing /aba/ frequently can induce adaptation, decreasing the likelihood of perceiving /aba/ in subsequent trials. We did not observe a reduction in ‘aba’ responses following auditory /aba/ trials compared to following auditory /ada/ trials (where neither adaptation nor recalibration is expected). Importantly, the reduction in ‘aba’ reports was specific to those trials following an illusory McGurk trial. Therefore, we believe that the increment in ‘ada’ responses after illusory McGurk trials can only be accounted for by recalibration and not by a process of selective speech adaptation process. We speculate that the absence of adaption effects in our experiment might be due to the use of a rapid recalibration design, where the adaptors randomly varied in each trial. Whereas assimilative effects can take place with just one trial of conflict exposure, contrastive effects usually require longer exposure periods than a single trial to build up [22,38].

## 5. Conclusion

Phonetic recalibration (i.e. a remapping of how a speech sound is perceived) depends on the perceptual interpretation of audiovisual information. Mere exposure to mismatching audiovisual speech (as during an non-fused incongruent McGurk video) is not sufficient to elicit recalibration. Instead, the inputs (auditory /aba/ and visual /aga/ in this case) have to be integrated to one percept (ada). Only then is there recalibration, which is manifested in a perceptual shift of the speech sound towards the perceptual interpretation. In a sense, not a mismatch between the two sensory modalities is key but a mismatch between the auditory input (/aba/) and the perceptual interpretation (ada). By recalibrating how /aba/ is perceived, the input is brought closer to the percept during a McGurk illusion and thereby the mismatch between input and percept is reduced. It would be interesting to see how far the requirement to successfully integrate multi-sensory events applies to other forms and modalities of multi-sensory integration, like visuo-proprioceptive [39] or audio-tactile [40] perception. Furthermore, the more similar two consecutively encountered sounds are, the stronger the remapping is. It gives an idea how the brain might be able to update the interpretation of multiple speaker-specific pronunciations in parallel, namely by shifting the representations of a specific speech sound independently of others.

## Supplementary Material

Supplemental Information: Rapid recalibration of speech perception after experiencing the McGurk illusion

## Data accessibility

The Donders Repository containing the project data can be accessed through the following link (persistent identifier): http://hdl.handle.net/11633/di.dccn.DSC_3018011.02_607.

(Prior to accessing and downloading the shared data, users must create an account. It is possible to use an institutional account or a social ID from Google+, Facebook, Twitter, LinkedIn or Microsoft. After authentication, users must accept the Data Use Agreement (DUA), after which they are automatically authorized to download the shared data. The DUA specifies whether there are any restrictions on how the data may be used).
