# Supplementary material for: Rapid recalibration of speech perception after experiencing the McGurk illusion
Source: R Soc Open Sci. 2018 Mar 28;5(3):170909. doi: 10.1098/rsos.170909 (PMC5882667; doi:10.1098/rsos.170909)
Supplement: Supplemental Information: Rapid recalibration of speech perception after experiencing the McGurk illusion [file rsos170909supp1.pdf]

Supplemental Information:
Rapid recalibration of speech perception after experiencing the McGurk illusion

Claudia S. Lüttke, Alexis Pérez-Bellido and Floris P. de Lange

Supplemental Information Inventory:

1. Supplemental Results
2. Supplemental Figure S1.
3. Supplemental Table S2.
4. Supplemental References.

Supplemental Results

Persistence of phonetic recalibration depends on the percept after the illusion

We tested whether the persistence of the recalibration effect was sensitive to the percept after the McGurk illusion, in other words whether recalibration lasted longer if two consecutive /aba/ sounds were misperceived as 'ada'. We compared the percentage of 'ada' percepts after recalibration occurred (/aba/ misperceived as 'ada' following a McGurk illusion; *recalibrated /aba/*) with the percentage of 'ada' percepts after recalibration did not occur (/aba/ perceived as 'aba' following a McGurk illusion; *non-recalibrated /aba/*). Additionally, we included the percentage of 'ada' percepts after participants correctly perceived /ada/ after a McGurk illusion in the analysis to account for priming of the 'ada' percept. To increase the number of observations per participant we averaged over same noise level on the previous and the current trial. We selected the *"same noise"* conditions, which according to our previous analyses maximize the recalibration effect. Only 40 of the 54 participants had enough trials (on average 18 trials per condition; SD = 6.1) in order to run our statistical tests (Figure S1). A repeated measures ANOVA on the percentage of 'ada' percepts, including condition on the previous trial (recalibrated /aba/, non-recalibrated /aba/ and /ada/) and condition on the current trial (/aba/ or /ada/) as within subjects factors, showed a main effect of condition on the previous trial ($F(1,39)=22.92$; $p=1.4e-08$), condition on the current trial ($F(1,39)=489.64$; $p=1.1e-23$), and an interaction between both factors ($F(2,78)=44.17$; $p=1.4e-13$). Specifically we observed that participants categorized /aba/ as 'ada' more often following a recalibrated /aba/ than following a non-recalibrated /aba/ ($t(39)= 8.62$, $p=1.51e-10$) or an /ada/ ($t(39)= 4.36$, $p=8.96e-5$). We also found a difference for how consecutive /ada/ trials were perceived. After a recalibrated /aba/ participants categorized /ada/ less often as 'ada' / than a after /ada/ ($t(39)= 3.91$, $p=3.5e-5$). We did not find a significant difference between the percentage of 'ada' after a non-recalibrated and recalibrated /aba/. This result suggests that experiencing a non-recalibrated /aba/ or a veridical /ada/ percept after a McGurk illusion, the recalibration effect diminishes due to the encountered new evidence. It provides additional support to the update belief model. That is, if after experiencing the illusion the new incoming evidence is veridically interpreted (i.e. /b/ perceived as 'b'), the prior /b/ distribution shifts back toward its original /b/ mapping.

Supplemental Figure

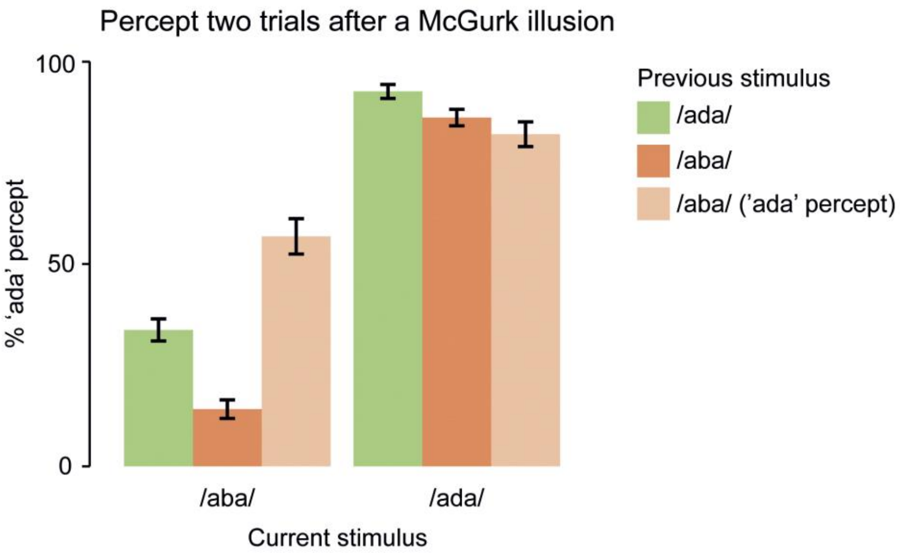

Figure S1. Persistence of the recalibration effect. The average percentage of 'ada' percepts during auditory /aba/ and /ada/ trials are shown here two trials after a McGurk illusion (N=40). The colors of the bars indicate the condition of the previous trial: /ada/ (green), non-recalibrated /aba/ (perceived as 'aba', dark orange), and recalibrated /aba/ (perceived as 'ada', light orange). Data represented only included averaged percentages of same levels of noise (between previous and current trial) conditions. Error bars display the standard errors of the mean.

Supplemental Table

Table S2. Probabilities of trial occurrences. The average number of trials occurring after each other is depicted below (previous trial in rows, current trial in columns). Since participants had one of five different trial orders the number of trials varies slightly across participants. Therefore, the average number of trials is not a whole number. In this study the analysis was restricted to auditory trials that were preceded by auditory or McGurk trials (grey). Trials occurring after a break were not included in the analysis and are not taking into account in the average below. Congruent audiovisual /aba/ and /ada/ trials were introduced as filler items and therefore occurred less frequently.

			**Trial n**										
Trial n-1			**Auditory**				**Audiovisual**						
			Aba		Ada		McGurk		Aba		Ada		
			low noise	high noise	low noise	high noise	low noise	high noise	low noise	high noise	low noise	high noise	∑
Auditory	Aba	low noise	24.6	24.4	24.2	23.4	22.8	23.6	3.6	3.0	2.6	4.0	*156.2*
		high noise	23.6	24.6	24.4	25.0	26.0	21.8	3.2	3.0	3.8	2.0	*157.4*
	Ada	low noise	24.8	25.2	23.0	25.0	26.4	20.2	3.4	2.4	3.2	3.2	*156.8*
		high noise	23.4	22.2	24.6	24.0	25.0	26.6	2.6	3.2	2.4	2.8	*156.8*
Audiovisual	McGurk	low noise	24.6	25.4	24.6	24.2	20.2	25.8	3.0	3.0	3.2	3.6	*157.6*
		high noise	24.4	24.4	24.6	23.8	23.4	26.8	2.6	2.4	2.2	2.2	*156.8*
	AvAba	low noise	2.8	2.8	1.8	3.2	3.4	2.8	0.6	0.6	0.6	0.8	*19.4*
		high noise	3.8	3.4	2.2	2.2	2.8	3.2	0.2	0.8	0.8	0.2	*19.6*
	AvAda	low noise	2.4	3.0	3.6	3.0	3.0	2.6	0.4	1.0	0.4	0.6	*20.0*
		high noise	2.8	2.2	3.8	2.8	2.8	3.2	0.4	0.6	0.6	0.2	*19.4*
		∑	*157.2*	*157.6*	*156.8*	*156.6*	*155.8*	*156.6*	*20.0*	*20.0*	*19.8*	*19.6*	*1020*

Supplemental References

Macmillan, N. A. & Creelman, C. D. *Detection Theory: A User's Guide*. (Lawrence Erlbaum Associates, 2005).
